# Molecular Mechanisms Employed by Neurons to Receive and Transduce Signals Essential for Learning and Memory

**DOI:** 10.3390/ijms24010206

**Published:** 2022-12-22

**Authors:** Antonis Stamatakis, Efthimios M. C. Skoulakis

**Affiliations:** 1 Department of Basic Sciences, School of Health Sciences National, Faculty of Nursing, Kapodistrian University of Athens, 11527 Athens, Greece; 2 Institute for Fundamental Biomedical Research, Biomedical Sciences Research Center “Alexander Fleming”, 16672 Vari, Greece

The ability to learn from the consequences of one’s actions, extracting useful information from threatening, painful or rewarding encounters and developing the skill of predicting probable events from pre-experienced stimuli, is essential for survival and reproductive success. To date, little is understood about the differential ways circuitry and molecular mechanisms may be engaged, or the part played by distinct types of learning and memory forms (e.g., episodic, emotional, procedural, working, and non-associative). Moreover, while intense study has examined the role of neurotransmitters and neuromodulators and the intracellular signalling pathways engaged, much remains unexplored.

Learning and memory comprise distinct processes, such as encoding, consolidation, storage, retrieval and the modulation of the mnemonic engram. Although the cellular and molecular events underlying these processes have been extensively studied, at present, understanding of the precise mechanisms differentiating them and the neural codes employed, are fragmentary at best. Considering the essential nature of learning and memory, another long-standing question is whether redundant but distinct molecular mechanisms subserve a particular form of learning and memory. Such redundancy would ensure that a measure of the capacity to acquire, integrate and consolidate information remains, even if inactivating mutations and/or circuit failures impair them.

This notion appears to agree with the ever-expanding list of novel cellular and biochemical functions and mechanisms that have been implicated in these processes and with newly uncovered functions of neurotransmitters already known to be engaged in these processes.

The aim of this Special Issue is to expand on the novel molecular mechanisms and circuits facilitating types of learning and memory in vertebrates and invertebrates. Το that end, the report by Lalo et al. demonstrates that surprisingly, in mammals, autophagy (a known mediator of proper diet and exercise on brain function and for neuroprotection from potentially harmful metabolites) also contributes to the maintenance of the excitatory/inhibitory balance, as well as to the synaptic plasticity in the neocortex and hippocampus, a prerequisite for memory encoding and consolidation. The effects of diet and metabolism on information acquisition in Drosophila are highlighted by the contribution of Myers et al. The work demonstrates that cholesterol homeostasis is essential for biogenic amine signalling, which supports efficient rates of aversive associative learning, and that the oldest known Drosophila mutant *white,* presents a learning rate deficit. Continuing the work on the novel functions of biogenic amines, Samaddar et al. present a highly novel role for serotonin in the development of the mammalian hippocampus. Interestingly, serotonin, through its 5-HT_1A_ receptors and downstream pathways involving PKCε and ERK1/2, increases neuronal precursor cell proliferation in the hippocampus during early postnatal development, in essence modulating the resultant number of resident mature neurons.

Regarding memory mechanisms and circuits, a shift in the accepted view is proposed and supported by the review by Dixsaut and Gräff. The medial prefrontal cortex, which had garnered little attention until recently, has emerged as a major hub for multiple stages of fear memory formation. In addition to its well-known role in long-term storage, it also appears to be a critical hub for encoding and consolidation. These novel functions of the medial prefrontal cortex are ostensibly a teaser for many more discoveries regarding this unique area of the vertebrate brain. For invertebrates, on the other hand, Bourouliti and Skoulakis demonstrate that a novel form of protein-synthesis-independent aversive memory in Drosophila actually comprises two different memories: one sensitive to amnestic treatment during its slower consolidation process, and another which consolidates faster and appears resistant to amnestic cold shock treatment. In a related review, the same authors summarise behavioural and molecular evidence from both vertebrates and invertebrates regarding the conservation and role of this massed-conditioning produced protein-synthesis-independent memory. In particular, they underscore the molecular evidence suggesting that this protein-synthesis-independent memory in Drosophila resembles fear memory in vertebrates, and likely depends on actin cytoskeletal dynamics for its consolidation.

This Special Issue highlights the expectation that many more surprising and novel findings are in store as the complex brain networks and molecular mechanisms that have evolved to ascertain survival in a complex and broadly unpredictable environment are revealed. The information is essential not only to begin understanding the organisation and function of our own brains, but also to model and build computational networks to facilitate the survival of our own species.

